# 291. Retrospective Study on Bacteremia in COVID-19 Patients in the Delta and Omicron Era

**DOI:** 10.1093/ofid/ofac492.369

**Published:** 2022-12-15

**Authors:** Sunghee Park, Tark Kim, Eun Ju Choo

**Affiliations:** Soonchunhyang University Bucheon Hospital, Bucheon, Kyonggi-do, Republic of Korea; Soonchunhyang University Bucheon Hospital, Bucheon, Kyonggi-do, Republic of Korea; Soonchunhyang University Bucheon Hospital, Bucheon, Kyonggi-do, Republic of Korea

## Abstract

**Background:**

The coronavirus disease 2019 (COVID-19) outbreak reached peak levels in South Korea as the Delta variant, dominant from late August until the end of 2021, was rapidly overtaken by the Omicron variant at the start of 2022. In studies conducted near the start of the pandemic, the occurrence of bacteremia in COVID-19 patients was relatively low. We aimed to determine if there was a change in the rate of bacteremia in COVID-19 patients with the progression of the pandemic.

**Methods:**

We performed a retrospective study of patients who were diagnosed with COVID-19 at a referral hospital between September 2021 and March 2022. Blood culture results were recorded, along with demographic characteristics and clinical outcomes. Contamination was considered when a single blood culture was positive for coagulase-negative staphylococci (CoNS), *Corynebacterium* species, or *Bacillus* species. Clinically relevant bacteremia was defined as bacteremia due to clinically significant pathogens between 7 days before COVID-19 diagnosis to 14 days after diagnosis.

**Results:**

Among the 360 patients included in the study, 46 cases from 43 (11.9%) patients were considered to be clinically relevant bacteremia. *Enterococcus faecalis* (17.4%) and CoNS (17.4%) were the most common pathogens, followed by *Acinetobacter baumannii* (15.2%), *Staphylococcus aureus* (10.7%) and *Escherichia coli* (10.7%). The median number of days from COVID-19 diagnosis to identification of bacteremia was 2 days. There was no significant difference in the rate of bacteremia between the Delta (September-December 2021) and Omicron variant eras (January-March 2022) (12.2% vs. 11.7%, *P* = .88). In the subgroup analysis of patients who received more than 2 days of intensive care, there was no statistical difference in the rate of bacteremia (14.5% [9/62] in the Delta variant era vs. 16.9% [14/83] in the Omicron variant era; *P* = .70). Mortality was significantly higher in patients with clinically relevant bacteremia (48.8% vs. 19.2%, *P* < .001).

**Conclusion:**

Many COVID-19 patients had bacteremia in the Omicron variant era, especially in the intensive care unit. Clinicians should suspect bacterial co-infection when a COVID-19 patient is clinically aggravated.

**Disclosures:**

**All Authors**: No reported disclosures.

